# High Throughput qPCR Expression Profiling of Circulating MicroRNAs Reveals Minimal Sex- and Sample Timing-Related Variation in Plasma of Healthy Volunteers

**DOI:** 10.1371/journal.pone.0145316

**Published:** 2015-12-23

**Authors:** Catherine Mooney, Rana Raoof, Hany El-Naggar, Amaya Sanz-Rodriguez, Eva M. Jimenez-Mateos, David C. Henshall

**Affiliations:** 1 Department of Physiology and Medical Physics, Royal College of Surgeons in Ireland, Dublin, Ireland; 2 Department of Anatomy, Mosul Medical College, Mosul, Iraq; 3 Department of Neurology, Beaumont Hospital, Dublin, Ireland; West Virginia University, UNITED STATES

## Abstract

MicroRNAs are a class of small non-coding RNA that regulate gene expression at a post-transcriptional level. MicroRNAs have been identified in various body fluids under normal conditions and their stability as well as their dysregulation in disease opens up a new field for biomarker study. However, diurnal and day-to-day variation in plasma microRNA levels, and differential regulation between males and females, may affect biomarker stability. A QuantStudio 12K Flex Real-Time PCR System was used to profile plasma microRNA levels using OpenArray in male and female healthy volunteers, in the morning and afternoon, and at four time points over a one month period. Using this system we were able to run four OpenArray plates in a single run, the equivalent of 32 traditional 384-well qPCR plates or 12,000 data points. Up to 754 microRNAs can be identified in a single plasma sample in under two hours. 108 individual microRNAs were identified in at least 80% of all our samples which compares favourably with other reports of microRNA profiles in serum or plasma in healthy adults. Many of these microRNAs, including miR-16-5p, miR-17-5p, miR-19a-3p, miR-24-3p, miR-30c-5p, miR-191-5p, miR-223-3p and miR-451a are highly expressed and consistent with previous studies using other platforms. Overall, microRNA levels were very consistent between individuals, males and females, and time points and we did not detect significant differences in levels of microRNAs. These results suggest the suitability of this platform for microRNA profiling and biomarker discovery and suggest minimal confounding influence of sex or sample timing. However, the platform has not been subjected to rigorous validation which must be demonstrated in future biomarker studies where large differences may exist between disease and control samples.

## Introduction

MicroRNAs (miRNAs) are small endogenous, non-coding RNA molecules, 18–25 nucleotides in length, which regulate gene expression in a post-transcriptional manner by binding to the 3’ untranslated region (UTR) of mRNA [1]. A single miRNA may regulate the expression of many genes by destabilization of the target mRNA allowing for the miRNA regulation of most protein-coding genes [2–4]. Release 21 of MiRBase, the miRNA sequence database, contains 2588 mature human miRNA sequence entries [5].

The presence of miRNAs in human plasma, serum and microvesicles was first reported by several groups in 2008 [6–8] and since then extracellular miRNAs have been profiled in various other body fluids including urine, cerebrospinal fluid, tears, saliva and peritoneal fluid [9]. Alterations in miRNA profiles in plasma and serum have been shown to occur in a range of diseases including various cancers, cardiovascular disease, liver and kidney disease, sepsis and neurological disorders (reviewed in [10–12]). This has generated much enthusiasm recently for the search for circulating miRNAs as novel non-invasive biomarkers for early disease detection.

MiRNA are ideal biomarker candidates as they are accessible in plasma and serum using minimally invasive techniques and are inexpensive to quantify. Circulating miRNA expression changes can occur earlier than conventional biomarkers [13] and the expression profiles reported are often more informative and discriminatory than mRNA profiles [10]. Despite the fact that extracellular miRNAs are predominantly exosomes/microvesicles free [14] miRNAs are more stable than mRNA in plasma or serum as they are resistant to RNase digestion [6, 8] due to their association with circulating Ago2 complexes [14, 15]. They have also been shown to be stable despite being exposed to extreme temperature changes, repeated freeze-thaw cycles, extended storage and changes in pH [6].

There are a number of different options available for miRNA profiling: hybridization (microarray/nCounter), small RNA Sequencing (miRNAseq) and reverse transcription–quantitative PCR (RT-qPCR) [16, 17]. There are advantages and disadvantages to each method, for example: miRNAseq is expensive but it is the only platform that can identify novel miRNAs; qRT-PCR and microarrays are relatively inexpensive but are limited in the number of miRNA which can be detected; and qRT-PCR is the only method which is able to provide absolute quantification. The choice of platform will influence the experiments which can be performed and the results obtained making comparisons between studies difficult. A recent study compared the reproducibility, specificity, sensitivity and accuracy of hybridization methods, RT-qPCR and miRNAseq, across 12 platforms from 9 different vendors [17]. They found higher overall detection rates and better sensitivity for RT-qPCR versus hybridization platforms. Only a few of the platforms captured small expression changes and there was low concordance of differential expression between the methods. They note that all the methods have strengths and weaknesses and the correct platform must be chosen with the particular study goals in mind. For example, if the detection of miRNA in a low RNA body fluid is the goal then platforms with high sensitivity should be prioritised over accurate quantification of miRNAs. Another important consideration is the substantial variability in the amount of input RNA required between platforms with qRT-PCR and microarrays generally requiring smaller amounts of input RNA than sequencing [17].

Before we can confidently predict that a miRNA is differentially expressed between healthy and diseased individuals we need to be confident that the miRNA under investigation is stable in healthy controls. Concern has been expressed as to the effect of diurnal and day-to-day variation in miRNA profiles [7, 18], the effect of ageing [19], ethnic differences [20], sex [21, 22], smoking [23], exercise [24] and fasting [25], for example. Studies have shown that not all miRNAs are common between whole blood, serum, plasma and exosomes and differences between the extraction methodology, the analysis platform and quantification strategies used to measure circulating miRNAs can influence results [7, 8, 26]. Many circulating miRNAs are shared within disease states as well as between healthy individuals making the selection of a disease-specific sensitive and specific biomarker challenging.

Here we set out to establish a benchmark for the use of the QuantStudio 12K Flex OpenArray Real-Time PCR System from Life Technologies for circulating miRNA profiling. We profiled circulating miRNA from plasma samples of healthy human volunteers and compared expression levels in the morning and afternoon, over the course of one month, and between males and females. To the best of our knowledge this is the first study to catalogue the normal spectrum of circulating miRNAs in such a manner using the OpenArray qRT-PCR system. Inspection of the circulating miRNA profiles revealed that the vast majority of miRNAs are stable across time points and between male and female individuals. We conclude that the OpenArray qRT-PCR platform is suitable for circulating miRNA profiling and biomarker discovery.

## Materials and Methods

### Sample collection and plasma processing

The study was approved by the Research Ethics Committee of the Royal College of Surgeons in Ireland (REC-859b) and written informed consent was obtained from all participants. 20 healthy non-fasting male and female volunteers were recruited for the study divided into three groups (Table 1), spanning a number of different European nationalities and ethnic backgrounds, which were not taken into consideration when assigning to specific groups. The first group, the morning-afternoon (AM-PM) group, was used to check whether the timing of blood collection (morning or afternoon sampling) had an effect on miRNA profile in healthy volunteers. Blood was collected from volunteers at 2 time points on the same day, between 9:30 am and 10:30 am and between 4:00 pm and 5:00 pm. At each time point 5 ml of blood was drawn into K2EDTA tubes (BD Bioscience). The second group, the day-to-day group, was used to check for miRNA stability in healthy volunteers over a period of time. 5 ml of blood was collected on 4 days over a 1 month period, day 1, day 2, day 7 and day 28, between 9:30 am and 10:30 am. The third group, the male-female group, was used to check for differential expression of miRNAs between males and females. The subjects in this group were included in the previous two groups, but only the morning samples for the morning-afternoon group and the day 1 samples from the day-to-day group were used.

**Table 1:** Demographics.

Cohort	Male	Female	Age range (mean)
AM-PM	5	5	26–53 (35.9)
Day-to-Day	5	5	23–46 (33.1)
Male-Female	10	10	23–53 (34.5)

Number of healthy volunteers included in each group, age ranges and mean.

Within one hour after blood collection, plasma was prepared by centrifuging the tubes at 1300 x g, for 10 minutes, at 4°*C*. The supernatant was collected into an RNAase free tube and extra care was taken not to disturb the buffy coat which contains the white blood cells. A second centrifugation step was then performed at 1940 x g for 10 minutes at 4°*C* which produced a visible white pellet in the sample [27]. Plasma was then collected into 1.5 ml RNAase free eppendorfs and stored at −80°*C*. The level of haemolysis in the plasma samples was assessed by spectrophotometric analysis using a Nanodrop 2000 spectrophotometer. The absorbance at 414 nm was checked and a cut-off level of 0.25 was used to distinguish haemolysis free samples [28].

### RNA extraction

The total RNA in the samples was isolated using a miRCURY^™^ RNA isolation kit (Exiqon) according to the manufacturer protocol. An input volume of 200 *μ*l of plasma was used, membranized particles were lysed using lysis solution, and proteins were precipitated using precipitation solution. After a centrifugation step for 3 minutes at 11,000 x g, the supernatant was collected into collection tube, mixed with Isopropanol and then loaded into a spin-column which binds only the RNA. After centrifugation for 30 seconds at 11,000 x g at room temperature, 100 *μ*l and 700 *μ*l of wash solution I and II were added to the spin column respectively followed by a centrifugation for 2 minutes at 11,000 x g to dry the column membrane completely. The final purified RNA was eluted in 25 *μ*l RNAase free water. RNA concentration in the sample and the level of protein contamination was assessed by Nanodrop 2000 spectrophotometer.

### MiRNA expression profiling

The OpenArray reverse transcription reaction was performed according to the manufacturer’s protocol using 3 *μ*l of total RNA in a 4.5 *μ*l mix of 0.75 *μ*l Megaplex RT primer pools (human Pools A or B Cat No. 4444750) from Applied Biosystem, 1.5 *μ*M dNTPs with dTTPs, 75U Multiscribe Reverse Transcriptase, 1X RT Buffer, 1.5 *μ*M MgCl2, 1.8U RNAase inhibitor (RT kit Cat No 4366596, Applied Biosystems). Reverse transcription reaction was performed in Applied Biosystems thermal cycler (for cycle conditions see S1 Table).

To increase the quantity of desired cDNA before performing PCR and to significantly increase the ability to detect low abundance transcripts, a pre-amplification step was performed according to the manufacturer’s recommendation. 2.5 *μ*l RT product was mixed with 1X Megaplex PreAmp primers (10X Human Pool A and B Cat. No. 4444748, Applied Biosystems), 1X TaqMan PreAmp master mix (2X, Cat No. 4391128, Applied Biosystems) to a final volumn of 25 *μ*l. Pre-amplification reaction was performed in an Applied Biosystems thermal cycler (for cycle conditions see S2 Table).

PreAmp product was first diluted with 0.1X TE to a ratio of 1:40, 22.5 *μ*l of diluted PreAmp product was then added to same volume of 2X TaqMan OpenArray Real time PCR Master Mix (Cat No. 4462164, Applied Biosystems) in the 384-well OpenArray sample loading plate. The manufacturer’s protocol was followed and the OpenArray panels were automatically loaded by the OpenArray AccuFill System. Each panel enables the quantification of miRNA expression in 3 samples and up to 4 panels can be cycled simultaneously, allowing for the analysis of 12 samples on a QuantStudio 12K Flex Real-Time PCR system. 754 human miRNAs were amplified in each sample together with 16 replicates each of 4 internal controls (ath-miR159a, RNU48, RNU44 and U6 rRNA).

### Data analysis

**Fig 1:**
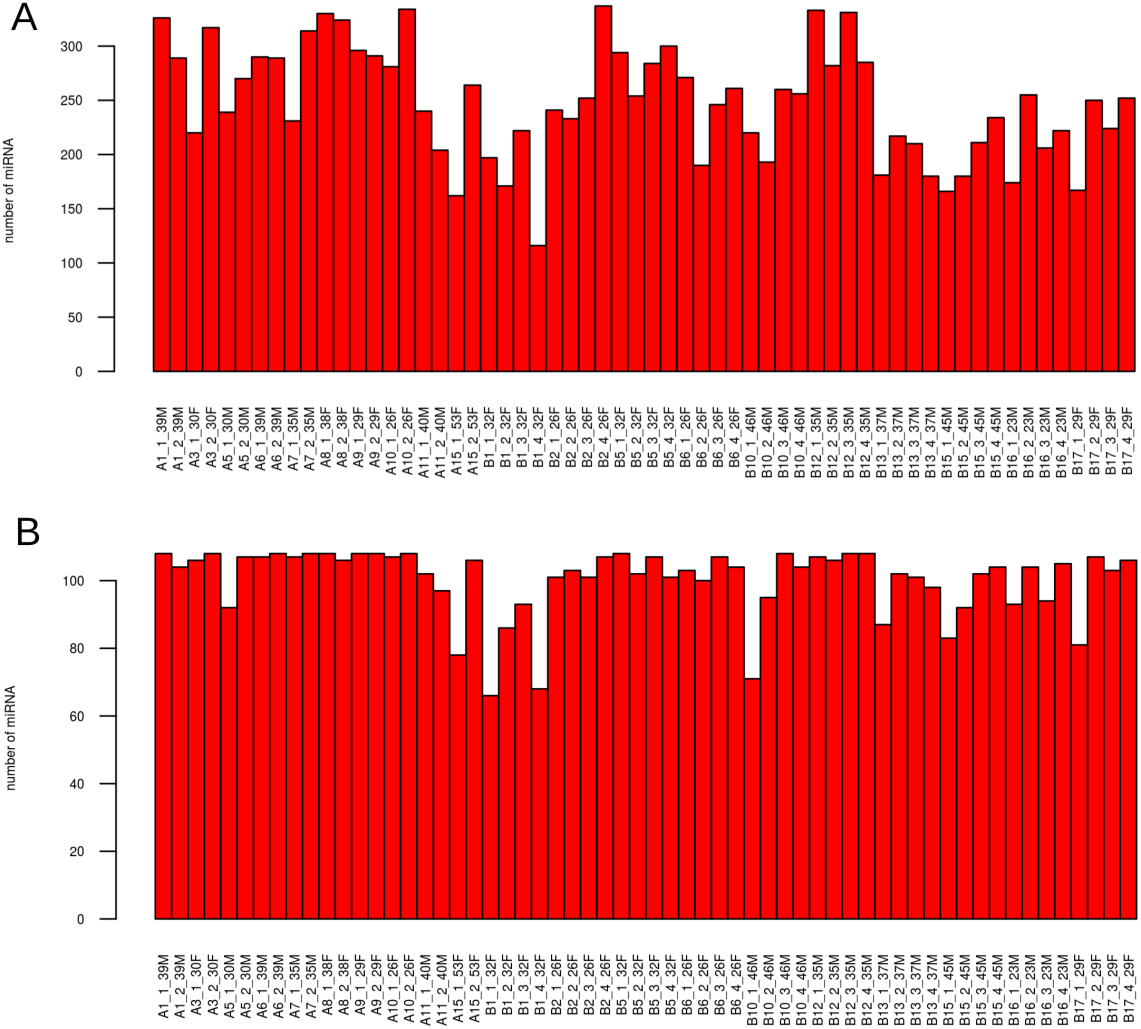
Bar plot showing the number of miRNA detected in samples. (a) before and (b) after filtering to remove any miRNA not present in 80% of samples.

All analyses were performed in R Bioconductor [29, 30]. The data was filtered as follows: if the cycle threshold (Ct) score for any miRNA in any sample was “Undetermined”; or if the quality control flags “AmpScore” or “CqConf” were < 1.24 or < .8 respectively; or if the Ct score was > 35, then the Ct score was set to 40. We then removed a miRNA if more than 20% of the individual observations were 40. Using these criteria, 646 miRNAs were filtered out leaving 108 miRNAs in the final data analysis including all samples (Fig 1 and S1 File). Missing data points (Ct = 40) were imputed (Bioconductor package “Non-detects” [31]) and the data was normalised using the DeltaCt method [32] as implemented in Bioconductor package “HTqPCR” [33]. The miRNA chosen to normalise the data were selected using a consensus between the top 10 most stable miRNA ranked by NormFinder [34] and geNorm [35], as implemented in Bioconductor package NormqPCR [36] (Table 2). *Δ*Ct was calculated by subtracting the mean of the Ct values of these miRNA from the Ct value of each miRNA, in each sample. Note that Ct values are inversely related to expression level i.e. a lower Ct value corresponds to higher expression.

**Table 2:** MiRNA to be used for normalisation.

NormFinder	geNorm
miR-15b-5p	miR-15b-5p
miR-17-5p	miR-17-5p
miR-24-3p	miR-24-3p
	miR-26b-5p
	miR-27a-3p
miR-30c-5p	miR-30c-5p
	miR-106a-5p
miR-126-5p	miR-126-5p
miR-1274b	
miR-223-3p	
miR-223-5p	
miR-331-3p	miR-331-3p
	miR-374a-5p
miR-532-3p	

The top 10 most stable miRNA in all samples as determined by NormFinder [34] and geNorm [35]. MiRNA common to both lists are used for normalisation.

Differential expression analysis was performed by applying a Student’s *t*-test to the normalized Ct values between the two conditions and the *p*-values were adjusted for multiple testing by controlling the false discovery rate (FDR) according to the method of Benjamini and Hochberg [37]. A miRNA was considered to be differentially expressed if the adjusted *p*-value was ≤ 0.05. The limma package [38] was used for analysis of the day-to-day samples.

## Results and Discussion

### Similarity to plasma profiles on other platforms

108 individual miRNAs were identified in at least 80% of all our samples (Fig 1). This compares favourable with other reports of miRNA profiles in serum or plasma in healthy adults: a group from Exiqon profiled miRNA from over 1500 serum and plasma samples and identified 119 miRNAs commonly found in serum and plasma [26]; 101 miRNAs were detected by Solexa sequencing of two pools of serum from 11 males and 10 females [6]; 106 and 118 miRNA were detected in all 6 plasma and serum samples respectively profiled on TaqMan human miRNA array panels [39]; and 90 and 99 miRNA were detected in all 10 plasma and serum samples respectively profiled on Exiqon human miRNA panels [39]. When only one sample, or a single pool of samples, is considered the reported number of miRNA identified in either plasma or serum tends to be higher, for example, 130 miRNA were identified in the plasma from a single healthy donor using TLDA array qRT-PCR profiling [8] and 349 miRNA were identified in the plasma of 5 pooled samples from healthy donors profiled using a miScript PCR System from Qiagen [9].

We checked to see the number of miRNA in common between these studies (Table 3) and also which miRNA in our set were identified most frequently in the other sets (Table 4). The Wang Exiqon list of miRNA is the most similar to any other set (75 in common with the Blondal Exiqon set) and also the least similar (46 Mooney OpenArray and 49 Weber Qiagen) (Table 3). 12 miRNAs in our set were not identified in any of the other sets: miR-30a-3p, miR-126-5p, miR-7-1-3p, miR-766-3p, miR-223-5p, miR-93-3p, miR-144-5p, miR-942-5p, miR-340-3p, miR-425-3p, miR-34a-3p and miR-1274b and 14 miRNA were found in all 7 sets: miR-19a-3p, miR-21-5p, miR-24-3p, miR-25-3p, miR-27b-3p, miR-30c-5p, miR-106b-5p, miR-148b-3p, miR-30b-5p, miR-451a, let-7g-5p, miR-223-3p, miR-191-5p and miR-17-5p (Table 4).

**Table 3:** Number of miRNA common between the seven sets of miRNA found in [6, 8, 26, 39, 39] and [9].

	Mooney OpenArray	Blondal Exiqon	Chen Solexa	Mitchell TaqMan	Wang Exiqon	Wang TaqMan	Weber Qiagen
Mooney OpenArray	-	68	52	53	46	67	61
Blondal Exiqon	68	-	73	61	75	69	66
Chen Solexa	52	73	-	58	62	53	61
Mitchell TaqMan	53	61	58	-	54	50	63
Wang Exiqon	46	75	62	54	-	52	49
Wang TaqMan	67	69	53	50	52	-	60
Weber Qiagen	61	66	61	63	49	60	-

OpenArray sequences and miRNA names in the other sets were mapped to the mature human sequences from miRBase version 21 [5]

**Table 4:** miRNA identified in 80% of our samples and their overlap with miRNA identified in five other studies of biofluids in healthy adults.

	Blondal [26]	Chen [6]	Mitchell [8]	Wang–Exiqon [39]	Wang–Taqman [39]	Weber [9]
miR-19a-3p	*	*	*	*	*	*
miR-21-5p	*	*	*	*	*	*
miR-24-3p	*	*	*	*	*	*
miR-25-3p	*	*	*	*	*	*
miR-27b-3p	*	*	*	*	*	*
miR-30c-5p	*	*	*	*	*	*
miR-106b-5p	*	*	*	*	*	*
miR-148b-3p	*	*	*	*	*	*
miR-30b-5p	*	*	*	*	*	*
miR-451a	*	*	*	*	*	*
let-7g-5p	*	*	*	*	*	*
miR-223-3p	*	*	*	*	*	*
miR-191-5p	*	*	*	*	*	*
miR-17-5p	*	*	*	*	*	*
miR-16-5p	*	*	*	*	*	
miR-19b-3p	*	*	*	*	*	
miR-27a-3p	*		*	*	*	*
miR-30d-5p	*	*	*	*		*
miR-92a-3p	*	*		*	*	*
miR-103a-3p	*	*	*	*		*
miR-142-3p	*	*	*	*	*	
miR-146a-5p	*	*	*	*	*	
miR-148a-3p	*	*	*	*		*
miR-192-5p	*	*	*		*	*
miR-221-3p	*		*	*	*	*
miR-301a-3p	*	*		*	*	*
miR-328-3p	*		*	*	*	*
miR-20a-5p	*	*	*	*	*	
miR-20b-5p	*	*	*		*	*
miR-93-5p	*	*	*		*	*
miR-29a-3p	*	*	*	*	*	
miR-324-3p	*		*	*	*	*
miR-106a-5p	*	*		*	*	*
miR-126-3p	*	*	*	*	*	
miR-222-3p	*	*	*	*	*	
let-7d-5p	*	*	*	*		*
miR-186-5p	*	*	*	*	*	
miR-199a-3p	*	*		*	*	*
miR-652-3p	*	*		*	*	*
miR-18a-5p	*	*	*		*	*
miR-15b-5p	*	*	*	*		
miR-26b-5p	*	*	*	*		
miR-130a-3p	*	*	*	*		
miR-130b-3p		*	*		*	*
miR-152-3p	*		*		*	*
miR-195-5p	*		*		*	*
miR-331-3p	*	*			*	*
miR-335-5p	*		*	*		*
miR-532-5p	*	*			*	*
miR-26a-5p		*	*	*		
miR-99b-5p	*		*		*	
miR-150-5p	*			*	*	
miR-324-5p	*	*			*	
miR-374a-5p		*			*	*
miR-375	*		*		*	
miR-376a-3p			*		*	*
miR-374b-5p	*	*				*
miR-590-5p		*			*	*
miR-345-5p			*		*	*
miR-122-5p	*			*	*	
miR-133a-3p	*				*	*
miR-320a	*			*	*	
miR-423-5p	*	*		*		
miR-574-3p	*			*	*	
miR-532-3p	*				*	*
miR-28-3p	*				*	*
let-7c-5p		*	*			
miR-28-5p	*				*	
miR-181a-5p	*	*				
miR-215-5p	*					*
miR-146b-5p		*			*	
miR-140-5p	*				*	
miR-410-3p			*			*
miR-487b-3p			*			*
miR-495-3p	*					*
miR-339-3p	*				*	
miR-128-3p	*					*
miR-143-3p	*					*
miR-340-5p		*	*			
miR-193a-5p					*	*
miR-885-5p					*	*
miR-483-5p					*	*
miR-155-5p			*		*	
miR-30a-5p						*
miR-30e-3p			*			
miR-127-3p					*	
miR-132-3p						*
miR-361-5p						*
miR-539-5p						*
miR-376c-3p					*	
miR-744-5p					*	
miR-409-3p						*
miR-193b-3p					*	
miR-628-3p						*
miR-590-3p						*

OpenArray sequences and miRNA names in the other sets were mapped to the mature human sequences from miRBase version 21 [5]

In conclusion, although there is a similar number of miRNA being detected across the studies examined here there is a large degree of variation between the lists of miRNA being detected by the different platforms, although we observered that there is a set of about 50 miRNA (Table 4) that are common to most of the studies most of the time. This would support the importance of independent validation of any potential biomarkers on another platform.

### Cellular origin of miRNA in plasma samples

Haider *et al.* [12] have created a miRNA expression matrix spanning 18 cell types, reflecting a broad range of most major cell types (epithelial, endothelial, mesenchymal, hematopoetic, and muscle). We examined the possible cellular origin of each of the 108 miRNA in our data set by cross checking them against the 100 most highly expressed miRNA in each of 18 unique cell types (Table 5).

**Table 5:** Possible cellular origin of miRNA identified in our plasma samples. a—acinar cell; b—adipocyte; c—ductal cell; d—endothelial; e—epithelial cell; f—fibroblast; g—hepatocyte; h—lymphatic EC; i—myocyte; j—neutrophil; k—smooth muscle cell; l—centroblast; m—memory B cell; n—monocyte; o—naïve B cell; p—NK cell; q—plasma B cell; and r—red blood cell. An asterix is plased in the column if the miRNA is found in the top 100 of miRNA expressed in that cell type. Expression profiles for all cells taken from [12]. miRNA are included if they are identified in at least one cell type.

**Non-Hematopoietic**												**Hematopoietic**						
	a	b	c	d	e	f	g	h	i	j	k	l	m	n	o	p	q	r
let-7c-5p	*	*	*	*	*	*	*	*	*	*	*	*	*	*	*		*	*
miR-15b-5p	*	*	*	*	*	*	*	*	*	*	*	*	*	*	*	*	*	*
miR-16-5p	*	*	*	*	*	*	*	*	*	*	*	*	*	*	*	*	*	*
miR-19a-3p	*	*	*	*	*	*	*	*	*	*		*	*	*	*	*	*	*
miR-19b-3p	*	*	*	*	*	*	*	*	*	*	*	*	*	*	*	*	*	*
miR-21-5p	*	*	*	*	*	*	*	*	*	*	*	*	*	*	*	*	*	*
miR-24-3p	*	*	*	*	*	*	*	*	*	*	*	*	*	*	*	*	*	*
miR-25-3p	*	*	*	*	*	*	*	*	*	*	*	*	*	*	*	*	*	*
miR-26a-5p	*	*	*	*	*	*	*	*	*	*	*	*	*	*	*	*	*	*
miR-26b-5p	*	*	*	*	*	*	*	*	*	*	*	*	*	*	*	*	*	*
miR-27a-3p	*	*	*	*	*	*	*	*	*	*	*	*	*	*	*	*	*	*
miR-27b-3p	*	*	*	*	*	*	*	*	*		*	*		*	*	*		
miR-28-5p	*	*	*	*	*		*				*	*	*	*	*		*	
miR-30a-3p	*			*			*	*			*							
miR-30a-5p	*	*	*	*	*	*	*	*	*		*	*					*	
miR-30c-5p	*		*	*	*	*	*	*	*	*		*	*	*	*	*	*	*
miR-30d-5p	*	*	*	*	*	*	*	*	*			*	*	*	*	*	*	*
miR-30e-3p	*				*		*		*			*	*	*	*		*	
miR-92a-3p	*	*	*	*	*	*	*	*		*	*	*	*	*	*	*	*	*
miR-99b-5p		*		*	*	*		*			*							
miR-103a-3p	*	*	*	*	*	*	*	*	*	*	*	*	*	*	*	*	*	*
miR-106b-5p	*	*	*	*	*	*	*	*	*	*	*	*	*	*	*	*	*	*
miR-126-5p				*				*						*				*
miR-127-3p		*		*		*		*		*	*							
miR-130a-3p	*	*	*	*	*	*	*	*	*		*			*				*
miR-130b-3p	*	*	*	*		*	*	*			*	*	*	*	*	*	*	*
miR-132-3p																*		
miR-142-3p	*		*							*		*	*	*	*	*	*	*
miR-146a-5p			*	*				*			*	*	*	*	*	*	*	
miR-148a-3p	*	*	*		*		*			*		*	*	*	*		*	*
miR-148b-3p	*			*	*		*					*	*	*	*	*	*	*
miR-150-5p			*									*	*	*	*	*	*	
miR-152-3p		*			*	*	*		*									
miR-181a-5p	*	*	*	*	*	*		*	*	*	*	*	*	*	*	*	*	*
miR-192-5p	*		*				*							*	*			*
miR-195-5p	*	*	*	*			*		*		*	*	*	*	*		*	*
miR-215-5p	*		*				*											*
miR-221-3p	*	*	*	*	*	*	*	*	*	*	*	*	*	*	*	*	*	
miR-301a-3p		*			*							*		*			*	*
miR-324-5p		*		*	*	*		*	*		*	*		*		*		*
miR-328-3p										*								
miR-331-3p	*	*	*	*	*	*	*	*	*	*	*	*	*	*	*	*	*	*
miR-335-5p	*								*					*				
miR-361-5p	*	*	*	*	*	*	*	*	*		*	*	*	*	*	*	*	
miR-374a-5p	*	*	*	*	*	*	*	*	*			*	*	*	*	*	*	*
miR-375	*		*				*											
miR-376a-3p	*	*	*	*		*		*	*		*			*				
miR-20a-5p	*	*	*	*	*	*	*	*	*	*	*	*	*	*	*	*	*	*
miR-30b-5p	*	*	*	*	*	*	*	*	*	*		*	*	*	*	*	*	*
miR-20b-5p	*	*	*	*	*		*	*	*			*	*	*	*	*	*	*
miR-93-5p	*	*	*	*	*	*	*	*	*	*	*	*	*	*	*	*	*	*
miR-146b-5p	*	*	*				*						*	*	*	*	*	
miR-451a	*		*			*		*					*	*				*
miR-140-5p		*	*	*		*	*		*	*	*	*	*	*	*		*	*
miR-410-3p		*		*		*					*							
miR-487b-3p				*	*	*			*		*	*	*		*			
miR-539-5p										*								
miR-532-5p				*					*									*
miR-495-3p		*									*							
miR-590-5p		*			*							*		*	*			*
miR-766-3p						*				*	*	*	*	*	*	*	*	*
miR-29a-3p	*	*	*	*	*	*	*	*	*	*	*	*	*	*	*	*	*	*
miR-376c-3p	*	*	*	*		*		*	*		*			*				
miR-324-3p	*	*	*	*	*	*	*	*	*	*	*	*	*	*	*	*	*	*
miR-345-5p								*										
miR-128-3p					*				*		*	*	*	*	*	*	*	*
miR-126-3p	*		*	*			*	*			*			*		*		*
miR-122-5p							*			*								
miR-133a-3p									*		*							
miR-143-3p	*	*	*	*	*	*	*		*	*	*	*	*	*	*	*	*	*
miR-340-3p														*				
miR-222-3p		*	*	*	*	*	*	*	*	*	*	*	*	*	*	*	*	*
miR-320a	*	*	*	*	*	*	*	*	*		*	*	*	*	*	*	*	*
let-7g-5p	*	*	*	*	*	*	*	*	*	*	*	*	*	*	*	*	*	*
let-7d-5p	*	*	*	*	*	*	*	*	*	*	*	*	*	*	*	*	*	*
miR-186-5p				*	*		*	*				*	*	*	*	*	*	*
miR-223-3p	*		*					*		*	*	*	*	*	*	*	*	*
miR-425-3p										*	*							*
miR-17-5p	*	*	*	*	*	*	*	*	*		*	*	*	*	*	*	*	*
miR-409-3p				*		*					*							
miR-574-3p	*		*			*	*	*		*			*	*	*			
miR-652-3p										*			*	*	*	*		*
miR-193b-3p	*	*		*	*	*	*		*		*	*	*		*		*	
miR-18a-5p				*			*					*	*	*	*	*	*	*
miR-155-5p				*		*	*	*			*	*	*	*	*	*	*	

23 miRNAs in our set of 108 miRNAs were not found in any of the 18 cell types and 18 miRNAs were ubiquitously expressed: miR-15b-5p, miR-16-5p, miR-19b-3p, miR-21-5p, miR-24-3p, miR-25-3p, miR-26a-5p, miR-26b-5p, miR-27a-3p, miR-103a-3p, miR-106b-5p, miR-331-3p, miR-20a-5p, miR-93-5p, miR-29a-3p, miR-324-3p, let-7g-5p and let-7d-5p. 78 miRNA were found in at least one and 37 were found in all of the seven hematopoetic cell types (centroblast, memory B cell, monocyte, naive B cell, NK cell, plasma B cell and red blood cell). 13 miRNA were not found in any of the hematopoetic cell types (miR-30a-3p, miR-99b-5p, miR-127-3p, miR-410-3p, miR-495-3p, miR-133a-3p, miR-409-3p, miR-152-3p, miR-328-3p, miR-375, miR-539-5p, miR-345-5p and miR-122-5p), but had possible cellular origin in at least one of the other 11 cell types (Table 5).

### Reproducibility

**Fig 2:**
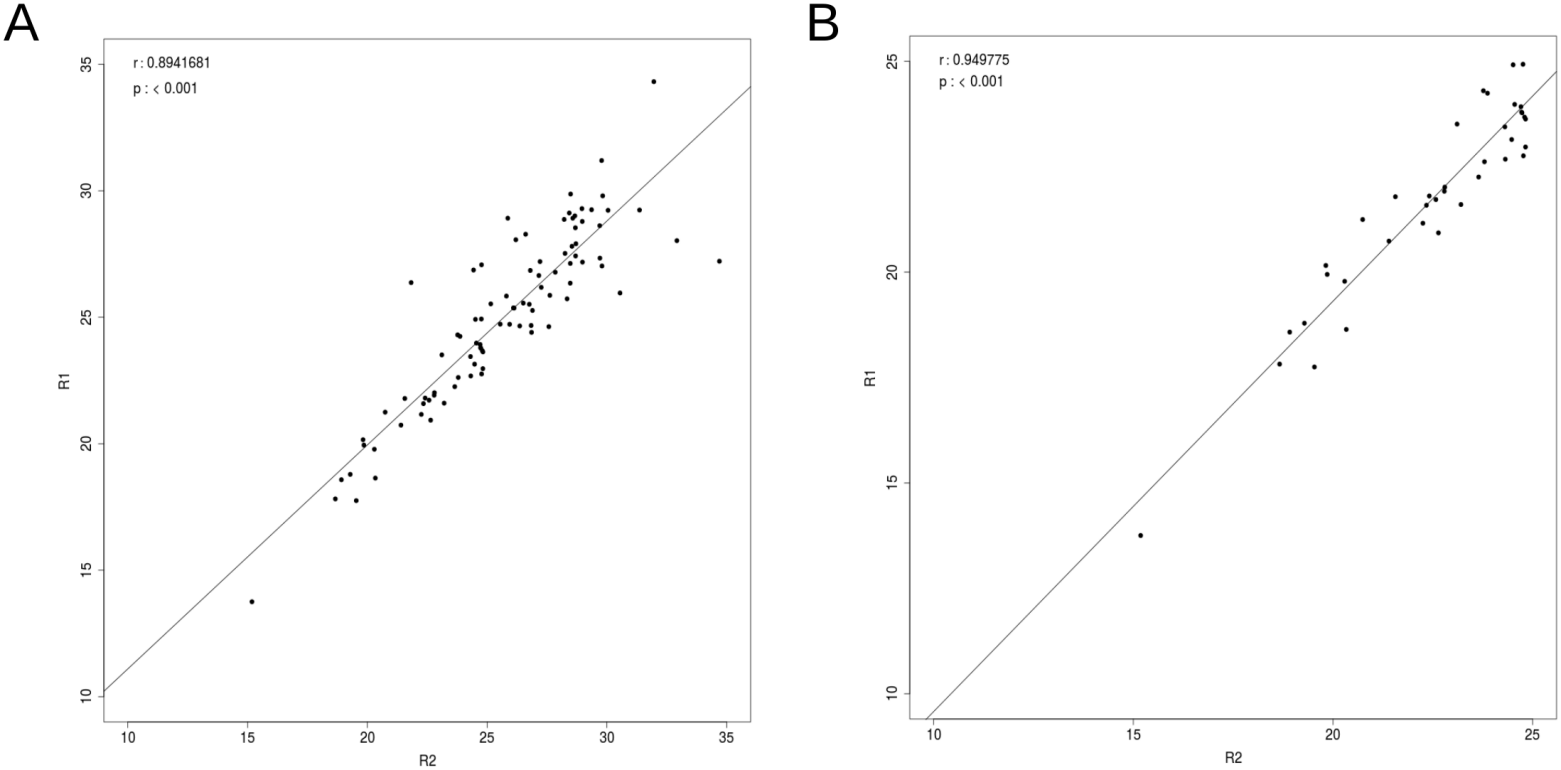
Reproducibility. Pairwise scatterplot of unnormalised data after filtering by CqConf and AmpScore, with no imputing of missing values. 122 miRNA were detected in sample R1 and 128 miRNA were detected in sample R2, 91 were common to both. (a) Ct > 35 are not included in the calculation of the correlation (b) Ct > 25 are not included in the calculation of the correlation.

To confirm the reproducibility of the OpenArray platform a blood sample was collected from a 28 year old male volunteer and prepared as described above. The RNA was extracted and the sample split into two parts (R1 and R2). Both parts were processed and profiled using the OpenArray system as previously described, the only difference being that the second part (R2) was frozen (at −80°*C*) for six months before processing and profiling. The data was filtering by CqConf and AmpScore as previously described but the data was not normalised and the missing values were not imputed. 122 miRNA were detected in sample R1 and 128 miRNA were detected in sample R2, 91 were common to both. A scatterplot of the Ct values in sample R1 against sample R2 showed a high degree of correlation between the Ct values in the two samples (Pearson’s correlation coefficient, *r* = 0.89), which increased when only high confidence Ct values, Ct ≤ 25, were included (*r* = 0.95) (Fig 2).

### Normalisation and differential expression analysis

The goal of normalisation is to remove nonspecific variation across samples that does not represent true biological difference related to the condition being studied by reducing technical error and variation and removing variation due to differences in RNA concentration between samples. This is especially critical when, as in this case, using a fixed volume of sample rather than a fixed quantity of RNA. However, there is no general consensus for the best method of normalisation [16, 40]. Wylie *et al.* compared seven methods for the normalisation of miRNA expression from biofluid and found that methods that focus on a restricted set of miRNAs tended to perform better than methods which focus on all miRNAs [41]. The choice of this set of miRNA is challenging as so called “housekeeping genes” which may be stable in a given cell type or experimental condition can vary considerably under disease conditions and between different tissues or biofluids, and no universally invariant miRNA or any other small RNA molecule has been found to date [9, 11, 25, 42].

**Fig 3:**
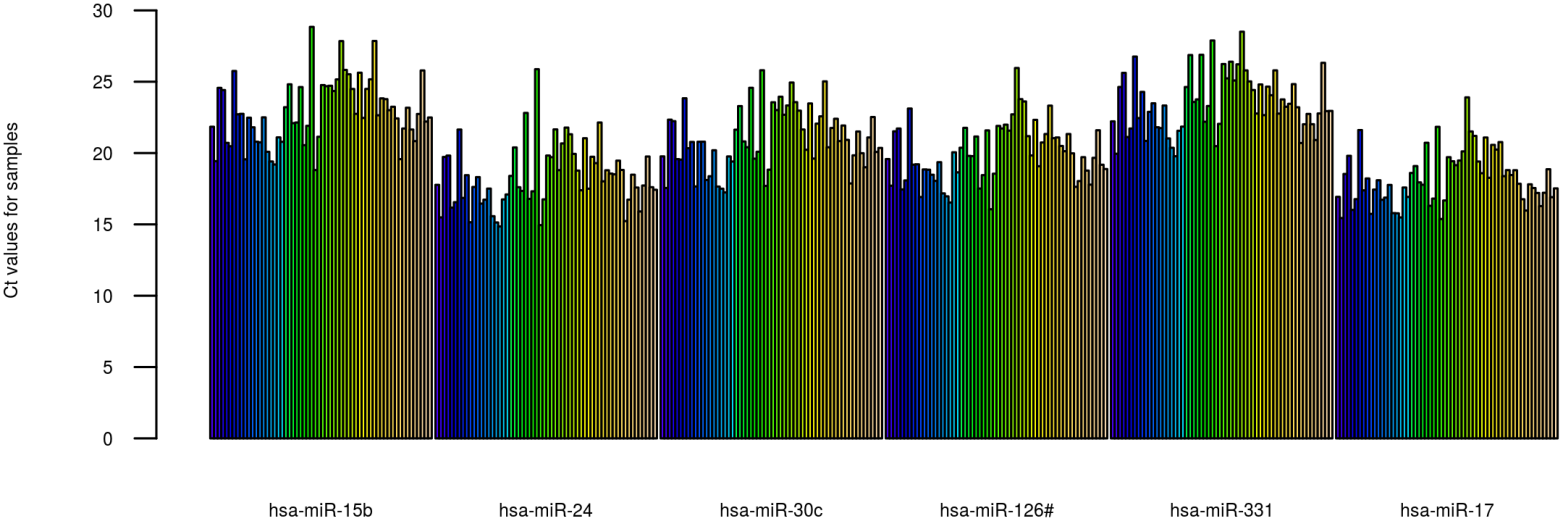
miRNA for normalisation. Bar plot showing the Ct values in each samples of the 6 miRNA chosen for normalisation.

For our analysis we have chosen a consensus between the top 10 most stable miRNA in all of our samples as determined by NormFinder [34] and geNorm [35] for normalisation. These are: miR-15b-5p, miR-17-5p, miR-24-3p, miR-30c-5p, miR-126-5p and miR-331-3p (Fig 3).

### Morning-afternoon group

It has been suggested that blood collection timing (day or night) might have an effect on miRNA expression [11]. This is important in the context of biomarker discovery especially if the disease in question has diurnal variations. A number of studies have provided evidence for the presence of rhythmically expressed miRNAs in various tissues [18, 43–47]. However, no studies have focused on the possibility of diurnal miRNA variation in blood sample collection. In the present study, to assess the possibility of morning-afternoon variation on miRNA profile, blood was collected from 10 healthy volunteers at two time points. A morning sample was collected between 9:30–10:30 am and an afternoon sample was collected between 4:00–5:00 pm. This group included 5 males with mean age 37 years (range 30–40 years) and 5 females with mean age 35 years (range 26–53 years). The samples were processed and the data analysed as previously described in Materials and Methods.

**Fig 4:**
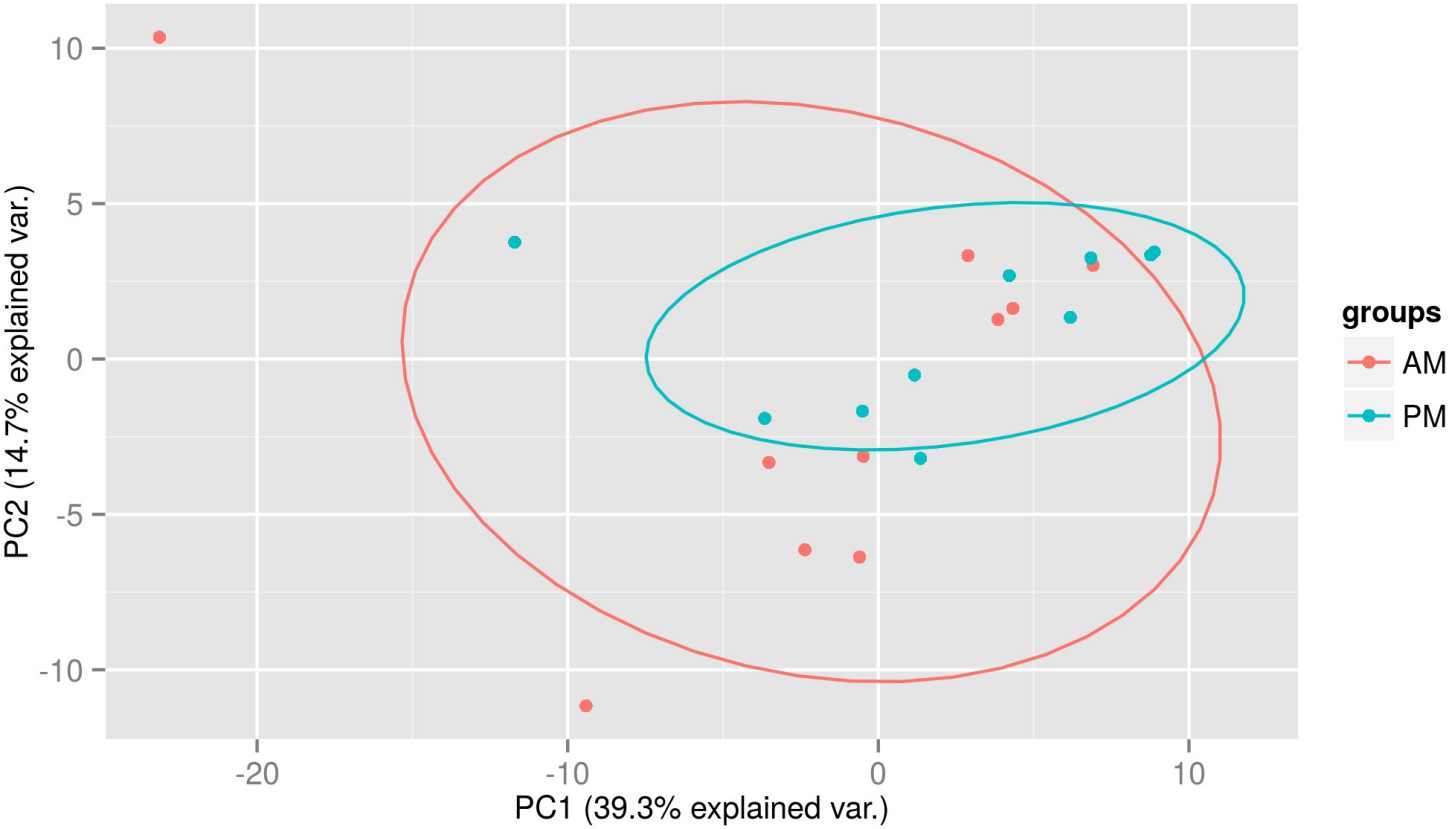
Principal component analysis. AM-PM—Samples taken from 5 females and 5 males, one morning sample and one afternoon sample from each is used (Plot created with R package ggbiplot).

Following normalisation we performed a principal component analysis and plotted the first two principal components but no clustering of the samples was observed (Fig 4). Similar to Moldovan *et al.* [11] we found no significantly differentially expressed miRNAs between the morning or afternoon samples. We repeated our analysis using two global normalisation methods, quantile normalisation [48] and normalisation to the geometric mean, as implemented in the Bioconductor package “HTqPCR” [33], however, we were still unable to identify any significantly differentially expressed miRNA. This suggests that timing of blood collection should not interfere with biomarker studies.

### Day-to-day group

To evaluate miRNA profile stability in healthy individuals over a period of time, blood was collected between 9:30 and 10:30 am, on 4 days over a one month period (day 1, day 2, day 7 and day 28). This group included 5 males with mean age 37 years (range 23–46) and 5 females with mean age 29 years (range 26–32 years). The samples were processed and the data analysed as previously described in Materials and Methods.

**Fig 5:**
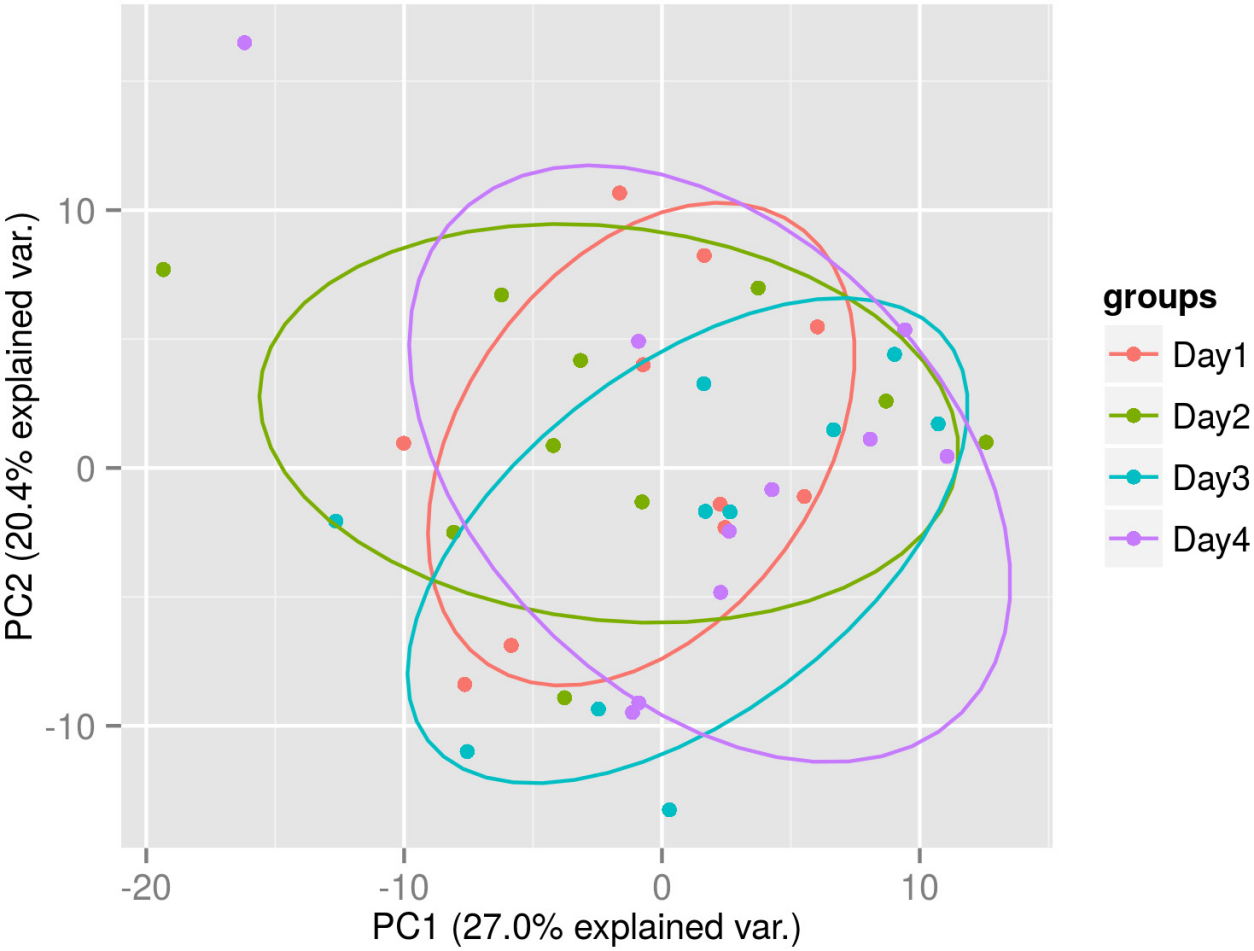
Principal component analysis. Day-to-Day—Samples taken from 5 females and 5 males on 4 mornings over a 1 month period, day 1, day 2, day 7 and day 28 (Plot created with R package ggbiplot).

**Fig 6:**
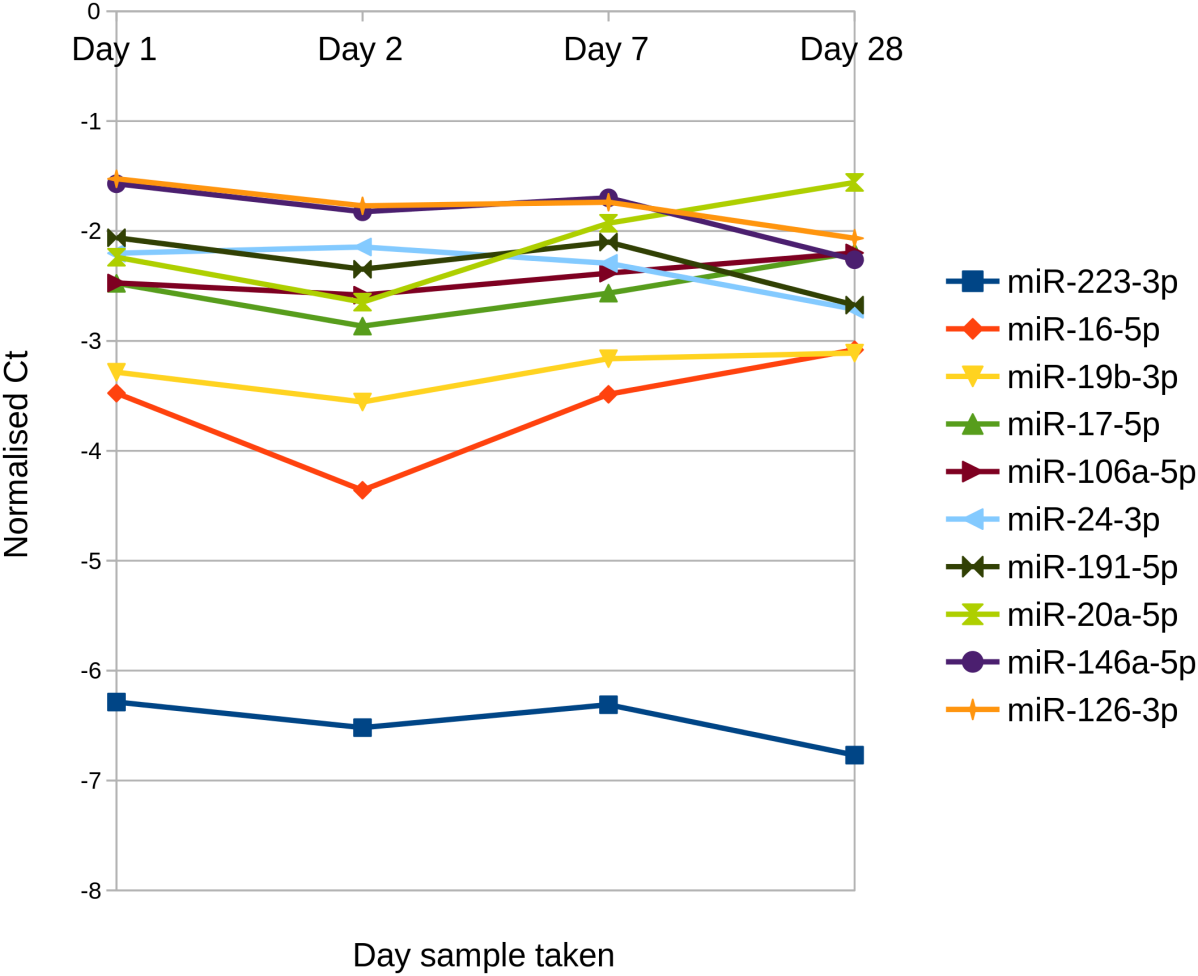
Top 10 most abundant miRNA in the day-to-day group.

All miRNAs screened were stable over this one month period, with no significant difference in expression between the four time points (see principal component analysis Fig 5). Fig 6 shows the variation in Ct values over the four sample times for the most abundant miRNA in these samples. As before we repeated our analysis using quantile normalisation and normalisation to the geometric mean, however, we still did not identify any differentially expressed miRNA. Although there was a slightly higher mean variance across the miRNA in the female samples compared to the male sample (2.15 in females, 1.8 in males), this was not found to be statistically significant. This is in agreement with MacLellan *et al.* [23] who found a high correlation (r = .88—.99) between pairs of serum samples taken from 12 healthy individuals between 2 and 17 months apart, and Rekker *et al.* [49] who found no significant differences in plasma miRNA expression levels in women during their menstrual cycle.

### Male-female group

To check for a possibility of miRNA differential expression between males and females, miRNA expression was analysed in 10 males with mean age 37 (range 23–46) and 10 females with mean age 32 (range 26–53). Blood was collected between 9:30 and 10:30 am. These subjects were included in the previous two groups. The samples were processed and the data analysed as previously described in Materials and Methods.

**Fig 7:**
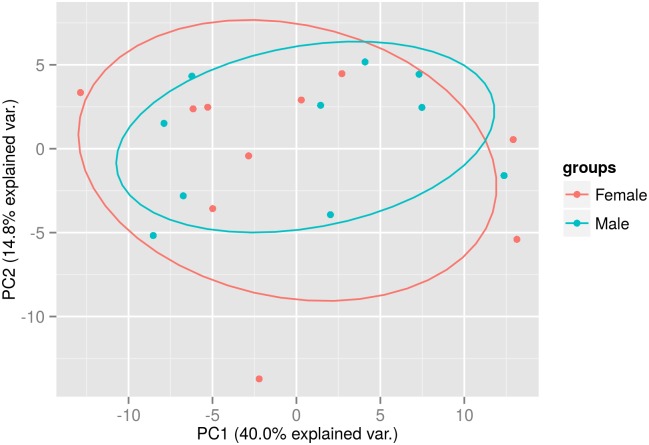
Principal component analysis. Male-Female—Samples taken from 10 females and 10 males, one morning sample from each individual is used (Plot created with R package ggbiplot).

**Fig 8:**
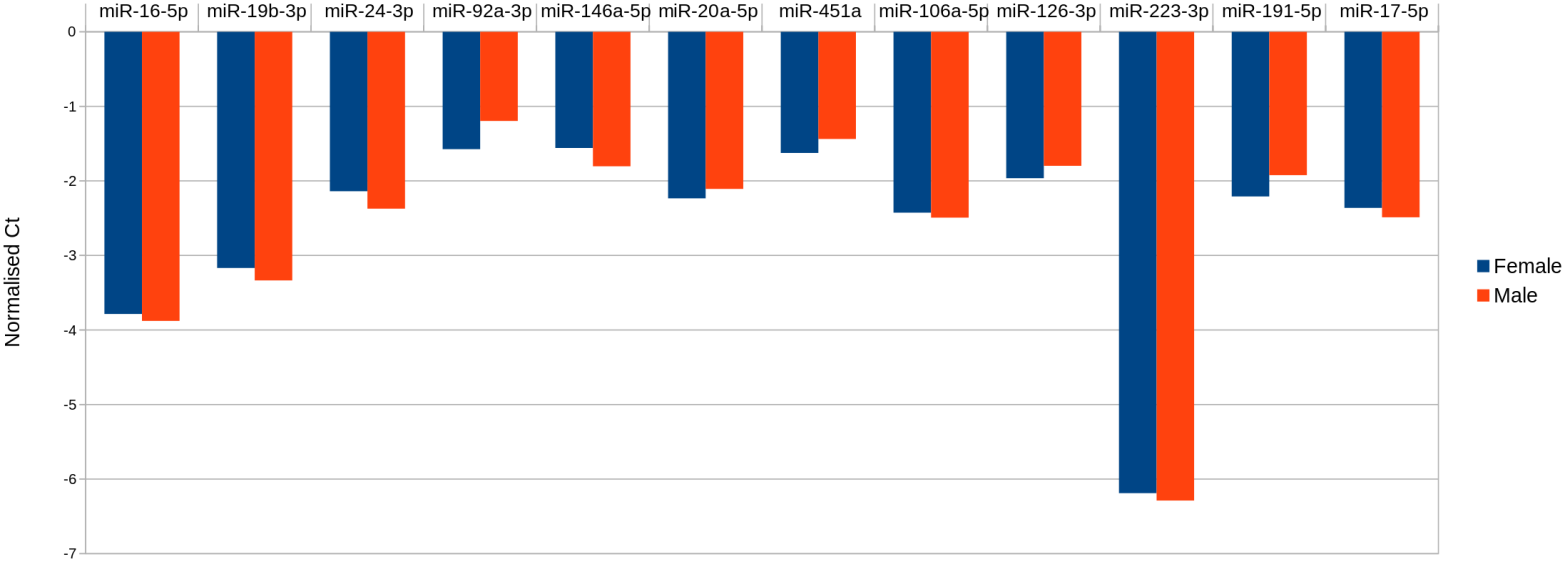
Top 12 most abundant miRNA in males and females.

Similar to Hunter *et al.* [7] we did not observe any significant difference between the male and female samples either after performing a principle component analysis (Fig 7) or differential expression analysis. Again, this did not change when we repeated our analysis using quantile normalisation and normalisation to the geometric mean. There was no significant difference in the number of miRNA detected in the male or female samples. There was slightly higher variance between miRNA detected in females than males (mean variance 1.94 and 1.79 respectively, after normalisation). We did not find any miRNA unique to either all male or female samples. The top 12 most abundant miRNA are the same for the male and female samples: miR-223-3p, miR-16-5p, miR-19b-3p, miR-106a-5p, miR-17-5p, miR-20a-5p, miR-191-5p, miR-24-3p, miR-126-3p, miR-451a, miR-92a-3p and miR-146a-5p (Fig 8).

However, other groups have detected differences. Four miRNAs (miR-548-3p, miR-1323, miR-940 and miR-1292) were found to be significantly up regulated in females by Duttagupta *et al.* [21] and one female specific miRNA (miR-222) and three male specific miRNAs (miR-100, miR-184, and miR-923) were identified in 11 male and 10 female pooled serum samples by Chen *et al.* [6]. miR-548-3p, miR-1323, miR-940, miR-1292 and miR-184, and miR-923 were not identified in our samples. We did however identify miR-222 in 9 out of 10 of both the female and male samples and miR-100 was identified in 5 female samples and 2 males samples, however there was no significant difference in expression between males and females in either case.

## Conclusion

Overall, miRNA levels were very consistent between individuals, males and females, and time points and miRNAs found to be highly expressed were consistent with previous studies of plasma from healthy controls using other platforms. These results would suggest the suitability of the QuantStudio 12K Flex OpenArray Real-Time PCR System for miRNA profiling and biomarker discovery. However, the platform has not yet been subjected to rigorous validation. The present study is not appropriate for this and therefore we cannot comment here on this aspect as we did not find differentially expressed miRNA between groups. In future biomarker studies where large differences may exist between disease and control samples the reproducibility and validation of the platform must be demonstrated.

## Supporting Information

S1 File Ct values for all samples. Ct values after filtering, imputing missing values and normalisation.
(CSV)

S1 Table Cycle conditions for the OpenArray reverse transcription reaction. (TIFF)

S2 Table Cycle conditions for the OpenArray pre-amplification reaction. (TIFF)

## References

[1] Bartel DP. MicroRNAs: genomics, biogenesis, mechanism, and function. Cell. 2004;116(2):281–297. 10.1016/S0092-8674(04)00045-5 14744438

[2] Friedman RC, Farh KKH, Burge CB, Bartel DP. Most mammalian mRNAs are conserved targets of microRNAs. Genome Research. 2009;19(1):92–105. 10.1101/gr.082701.108 18955434 PMC2612969

[3] Bartel DP. MicroRNAs: target recognition and regulatory functions. Cell. 2009;136(2):215–233. 10.1016/j.cell.2009.01.002 19167326 PMC3794896

[4] Guo H, Ingolia NT, Weissman JS, Bartel DP. Mammalian microRNAs predominantly act to decrease target mRNA levels. Nature. 2010;466(7308):835–840. 10.1038/nature09267 20703300 PMC2990499

[5] Kozomara A, Griffiths-Jones S. miRBase: annotating high confidence microRNAs using deep sequencing data. Nucleic Acids Research. 2014;42(D1):D68 10.1093/nar/gkt1181 24275495 PMC3965103

[6] Chen X, Ba Y, Ma L, Cai X, Yin Y, Wang K, et al Characterization of microRNAs in serum: a novel class of biomarkers for diagnosis of cancer and other diseases. Cell Research. 2008;18(10):997–1006. 10.1038/cr.2008.282 18766170

[7] Hunter MP, Ismail N, Zhang X, Aguda BD, Lee EJ, Yu L, et al Detection of microRNA expression in human peripheral blood microvesicles. PLoS One. 2008;3(11):e3694 10.1371/journal.pone.0003694 19002258 PMC2577891

[8] Mitchell PS, Parkin RK, Kroh EM, Fritz BR, Wyman SK, Pogosova-Agadjanyan EL, et al Circulating microRNAs as stable blood-based markers for cancer detection. Proceedings of the National Academy of Sciences. 2008;105(30):10513–10518. 10.1073/pnas.0804549105 PMC2492472 18663219

[9] Weber JA, Baxter DH, Zhang S, Huang DY, Huang KH, Lee MJ, et al The microRNA spectrum in 12 body fluids. Clinical Chemistry. 2010;56(11):1733–1741. 10.1373/clinchem.2010.147405 20847327 PMC4846276

[10] Reid G, Kirschner MB, van Zandwijk N. Circulating microRNAs: Association with disease and potential use as biomarkers. Critical Reviews in Oncology/Hematology. 2011;80(2):193–208. 10.1016/j.critrevonc.2010.11.004 21145252

[11] Moldovan L, Batte KE, Trgovcich J, Wisler J, Marsh CB, Piper M. Methodological challenges in utilizing miRNAs as circulating biomarkers. Journal of Cellular and Molecular Medicine. 2014;18(3):371–390. 10.1111/jcmm.12236 24533657 PMC3943687

[12] Haider BA, Baras AS, McCall MN, Hertel JA, Cornish TC, Halushka MK. A critical evaluation of microRNA biomarkers in non-neoplastic disease. PLoS One. 2014;9(2):e89565 10.1371/journal.pone.0089565 24586876 PMC3935874

[13] Ward J, Kanchagar C, Veksler-Lublinsky I, Lee RC, McGill MR, Jaeschke H, et al Circulating microRNA profiles in human patients with acetaminophen hepatotoxicity or ischemic hepatitis. Proceedings of the National Academy of Sciences. 2014;111(33):12169–12174. 10.1073/pnas.1412608111 PMC4143020 25092309

[14] Turchinovich A, Weiz L, Langheinz A, Burwinkel B. Characterization of extracellular circulating microRNA. Nucleic Acids Research. 2011;39(16):7223–7233. 10.1093/nar/gkr254 21609964 PMC3167594

[15] Arroyo JD, Chevillet JR, Kroh EM, Ruf IK, Pritchard CC, Gibson DF, et al Argonaute2 complexes carry a population of circulating microRNAs independent of vesicles in human plasma. Proceedings of the National Academy of Sciences. 2011;108(12):5003–5008. 10.1073/pnas.1019055108 PMC3064324 21383194

[16] Meyer SU, Pfaffl MW, Ulbrich SE. Normalization strategies for microRNA profiling experiments: a ‘normal’way to a hidden layer of complexity? Biotechnology Letters. 2010;32(12):1777–1788. 10.1007/s10529-010-0380-z 20703800

[17] Mestdagh P, Hartmann N, Baeriswyl L, Andreasen D, Bernard N, Chen C, et al Evaluation of quantitative miRNA expression platforms in the microRNA quality control (miRQC) study. Nature Methods. 2014;11(8):809–815. 10.1038/nmeth.3014 24973947

[18] Shende VR, Goldrick MM, Ramani S, Earnest DJ. Expression and rhythmic modulation of circulating microRNAs targeting the clock gene Bmal1 in mice. PLoS One. 2011;6(7):e22586 10.1371/journal.pone.0022586 21799909 PMC3142187

[19] Drummond MJ, McCarthy JJ, Sinha M, Spratt HM, Volpi E, Esser KA, et al Aging and microRNA expression in human skeletal muscle: a microarray and bioinformatics analysis. Physiological Genomics. 2011;43(10):595–603. 10.1152/physiolgenomics.00148.2010 20876843 PMC3110890

[20] Bovell LC, Shanmugam C, Putcha BDK, Katkoori VR, Zhang B, Bae S, et al The prognostic value of microRNAs varies with patient race/ethnicity and stage of colorectal cancer. Clinical Cancer Research. 2013;19(14):3955–3965. 10.1158/1078-0432.CCR-12-3302 23719259 PMC3746330

[21] Duttagupta R, Jiang R, Gollub J, Getts RC, Jones KW. Impact of cellular miRNAs on circulating miRNA biomarker signatures. PLoS One. 2011;6(6):e20769 10.1371/journal.pone.0020769 21698099 PMC3117799

[22] Morgan CP, Bale TL, et al Sex differences in microRNA regulation of gene expression: no smoke, just miRs. Biology of Sex Differences. 2012;3(1):22 10.1186/2042-6410-3-22 23009289 PMC3507674

[23] MacLellan SA, MacAulay C, Lam S, Garnis C. Pre-profiling factors influencing serum microRNA levels. BMC Clinical Pathology. 2014;14(1):27 10.1186/1472-6890-14-27 25093010 PMC4107491

[24] Baggish AL, Hale A, Weiner RB, Lewis GD, Systrom D, Wang F, et al Dynamic regulation of circulating microRNA during acute exhaustive exercise and sustained aerobic exercise training. The Journal of Physiology. 2011;589(16):3983–3994. 10.1113/jphysiol.2011.213363 21690193 PMC3179997

[25] Kroh EM, Parkin RK, Mitchell PS, Tewari M. Analysis of circulating microRNA biomarkers in plasma and serum using quantitative reverse transcription-PCR (qRT-PCR). Methods. 2010;50(4):298–301. 10.1016/j.ymeth.2010.01.032 20146939 PMC4186708

[26] Blondal T, Jensby Nielsen S, Baker A, Andreasen D, Mouritzen P, Wrang Teilum M, et al Assessing sample and miRNA profile quality in serum and plasma or other biofluids. Methods. 2013;59(1):S1–S6. 10.1016/j.ymeth.2012.09.015 23036329

[27] Cheng HH, Yi HS, Kim Y, Kroh EM, Chien JW, Eaton KD, et al Plasma processing conditions substantially influence circulating microRNA biomarker levels. PLoS One. 2013;8(6):e64795 10.1371/journal.pone.0064795 23762257 PMC3676411

[28] Kirschner MB, Kao SC, Edelman JJ, Armstrong NJ, Vallely MP, van Zandwijk N, et al Haemolysis during sample preparation alters microRNA content of plasma. PLoS One. 2011;6(9):e24145 10.1371/journal.pone.0024145 21909417 PMC3164711

[29] R Core Team. R: A Language and Environment for Statistical Computing. Vienna, Austria; 2015 Available from: http://www.R-project.org/.

[30] Huber W, Carey VJ, Gentleman R, Anders S, Carlson M, Carvalho BS, et al Orchestrating high-throughput genomic analysis with Bioconductor. Nature Methods. 2015;12(2):115–121. 10.1038/nmeth.3252 25633503 PMC4509590

[31] McCall MN, McMurray HR, Land H, Almudevar A. On non-detects in qPCR data. Bioinformatics. 2014;30(16):2310–2316. 10.1093/bioinformatics/btu239 24764462 PMC4133581

[32] Livak KJ, Schmittgen TD. Analysis of relative gene expression data using real-time quantitative PCR and the 2^−*ΔΔCt*^ method. Methods. 2001;25(4):402–408. 10.1006/meth.2001.1262 11846609

[33] Dvinge H, Bertone P. HTqPCR: High-throughput analysis and visualization of quantitative real-time PCR data in R. Bioinformatics. 2009;25(24):3325 10.1093/bioinformatics/btp578 19808880 PMC2788924

[34] Andersen CL, Jensen JL, Ørntoft TF. Normalization of real-time quantitative reverse transcription-PCR data: a model-based variance estimation approach to identify genes suited for normalization, applied to bladder and colon cancer data sets. Cancer Research. 2004;64(15):5245–5250. 10.1158/0008-5472.CAN-04-0496 15289330

[35] Vandesompele J, De Preter K, Pattyn F, Poppe B, Van Roy N, De Paepe A, et al Accurate normalization of real-time quantitative RT-PCR data by geometric averaging of multiple internal control genes. Genome Biology. 2002;3(7):research0034 10.1186/gb-2002-3-7-research0034 12184808 PMC126239

[36] Perkins JR, Dawes JM, McMahon SB, Bennett DL, Orengo C, Kohl M. ReadqPCR and NormqPCR: R packages for the reading, quality checking and normalisation of RT-qPCR quantification cycle (Cq) data. BMC Genomics. 2012;13(1):296 10.1186/1471-2164-13-296 22748112 PMC3443438

[37] Benjamini Y, Hochberg Y. Controlling the false discovery rate: a practical and powerful approach to multiple testing. Journal of the Royal Statistical Society Series B (Methodological). 1995;p. 289–300.

[38] Ritchie ME, Phipson B, Wu D, Hu Y, Law CW, Shi W, et al *limma* powers differential expression analyses for RNA-sequencing and microarray studies. Nucleic Acids Research. 2015;43(7):e47 10.1093/nar/gkv007 25605792 PMC4402510

[39] Wang K, Yuan Y, Cho JH, McClarty S, Baxter D, Galas DJ. Comparing the MicroRNA spectrum between serum and plasma. PLoS One. 2012;7(7):e41561 10.1371/journal.pone.0041561 22859996 PMC3409228

[40] Pradervand S, Weber J, Thomas J, Bueno M, Wirapati P, Lefort K, et al Impact of normalization on miRNA microarray expression profiling. RNA. 2009;15(3):493–501. 10.1261/rna.1295509 19176604 PMC2657010

[41] Wylie D, Shelton J, Choudhary A, Adai AT. A novel mean-centering method for normalizing microRNA expression from high-throughput RT-qPCR data. BMC Research Notes. 2011;4(1):555 10.1186/1756-0500-4-555 22188771 PMC3267743

[42] Mestdagh P, Van Vlierberghe P, De Weer A, Muth D, Westermann F, Speleman F, et al A novel and universal method for microRNA RT-qPCR data normalization. Genome Biology. 2009;10(6):R64 10.1186/gb-2009-10-6-r64 19531210 PMC2718498

[43] Cheng HYM, Papp JW, Varlamova O, Dziema H, Russell B, Curfman JP, et al microRNA modulation of circadian-clock period and entrainment. Neuron. 2007;54(5):813–829. 10.1016/j.neuron.2007.05.017 17553428 PMC2590749

[44] Na YJ, Sung JH, Lee SC, Lee YJ, Choi YJ, Park WY, et al Comprehensive analysis of microRNA-mRNA co-expression in circadian rhythm. Experimental & Molecular Medicine. 2009;41(9):638–647. 10.3858/emm.2009.41.9.070 19478556 PMC2753657

[45] Gatfield D, Le Martelot G, Vejnar CE, Gerlach D, Schaad O, Fleury-Olela F, et al Integration of microRNA miR-122 in hepatic circadian gene expression. Genes & development. 2009;23(11):1313–1326. 10.1101/gad.1781009 19487572 PMC2701584

[46] Chen R, D’Alessandro M, Lee C. miRNAs are required for generating a time delay critical for the circadian oscillator. Current Biology. 2013;23(20):1959–1968. 10.1016/j.cub.2013.08.005 24094851 PMC3809330

[47] Kinoshita C, Aoyama K, Matsumura N, Kikuchi-Utsumi K, Watabe M, Nakaki T. Rhythmic oscillations of the microRNA miR-96-5p play a neuroprotective role by indirectly regulating glutathione levels. Nature Communications. 2014;5 (3823). 10.1038/ncomms4823 PMC4024755 24804999

[48] Bolstad BM, Irizarry RA, Åstrand M, Speed TP. A comparison of normalization methods for high density oligonucleotide array data based on variance and bias. Bioinformatics. 2003;19(2):185–193. 10.1093/bioinformatics/19.2.185 12538238

[49] Rekker K, Saare M, Roost AM, Salumets A, Peters M. Circulating microRNA Profile throughout the Menstrual Cycle. PLoS One. 2013;8(11):e81166 10.1371/journal.pone.0081166 24244734 PMC3828277

