# Supplementary figures and images for: High Throughput qPCR Expression Profiling of Circulating MicroRNAs Reveals Minimal Sex- and Sample Timing-Related Variation in Plasma of Healthy Volunteers
Source: PLoS One. 2015 Dec 23;10(12):e0145316. doi: 10.1371/journal.pone.0145316 (PMC4689368; doi:10.1371/journal.pone.0145316)

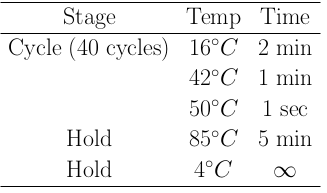

Supplement: S1 Table — (TIFF) [file pone.0145316.s002.tiff]

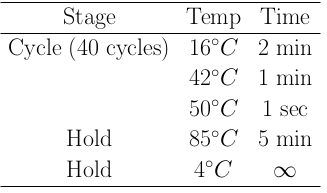

Supplement: S2 Table — (TIFF) [file pone.0145316.s003.tiff]
